# Inhibition of Murine Cytomegalovirus Infection in Animals by RNase P-Associated External Guide Sequences

**DOI:** 10.1016/j.omtn.2017.10.007

**Published:** 2017-10-16

**Authors:** Wei Li, Jingxue Sheng, Mengqiong Xu, Gia-Phong Vu, Zhu Yang, Yujun Liu, Xu Sun, Phong Trang, Sangwei Lu, Fenyong Liu

**Affiliations:** 1 Department of Biotechnology, College of Life Science and Technology, Jinan University, Guangzhou, Guangdong 510632, China; 2 Program in Comparative Biochemistry, University of California, Berkeley, Berkeley, CA 94720, USA; 3 School of Public Health, University of California, Berkeley, Berkeley, CA 94720, USA; 4 Jiangsu Affynigen Biotechnolgies, Inc., Taizhou, Jiangsu 225300, China; 5 Guangzhou Qinheli Biotechnolgies, Inc., Guangzhou, Guangdong 510600, China; 6 School of Medicine, St. George’s University, Grenada, West Indies; 7 School of Pharmacy, Shandong University of Traditional Chinese Medicine, Jinan, Shandong 250355, China

**Keywords:** antisense, gene therapy, herpesvirus, RNase P, gene targeting

## Abstract

External guide sequence (EGS) RNAs are associated with ribonuclease P (RNase P), a tRNA processing enzyme, and represent promising agents for gene-targeting applications as they can direct RNase-P-mediated cleavage of a target mRNA. Using murine cytomegalovirus (MCMV) as a model system, we examined the antiviral effects of an EGS variant, which was engineered using *in vitro* selection procedures. EGSs were used to target the shared mRNA region of MCMV capsid scaffolding protein (mCSP) and assemblin. *In vitro*, the EGS variant was 60 times more active in directing RNase P cleavage of the target mRNA than the EGS originating from a natural tRNA. In MCMV-infected cells, the variant reduced mCSP expression by 92% and inhibited viral growth by 8,000-fold. In MCMV-infected mice hydrodynamically transfected with EGS-expressing constructs, the EGS variant was more effective in reducing mCSP expression, decreasing viral production, and enhancing animal survival than the EGS originating from a natural tRNA. These results provide direct evidence that engineered EGS variants with higher targeting activity *in vitro* are also more effective in reducing gene expression in animals. Furthermore, our findings imply the possibility of engineering potent EGS variants for therapy of viral infections.

## Introduction

**Figure 1:**
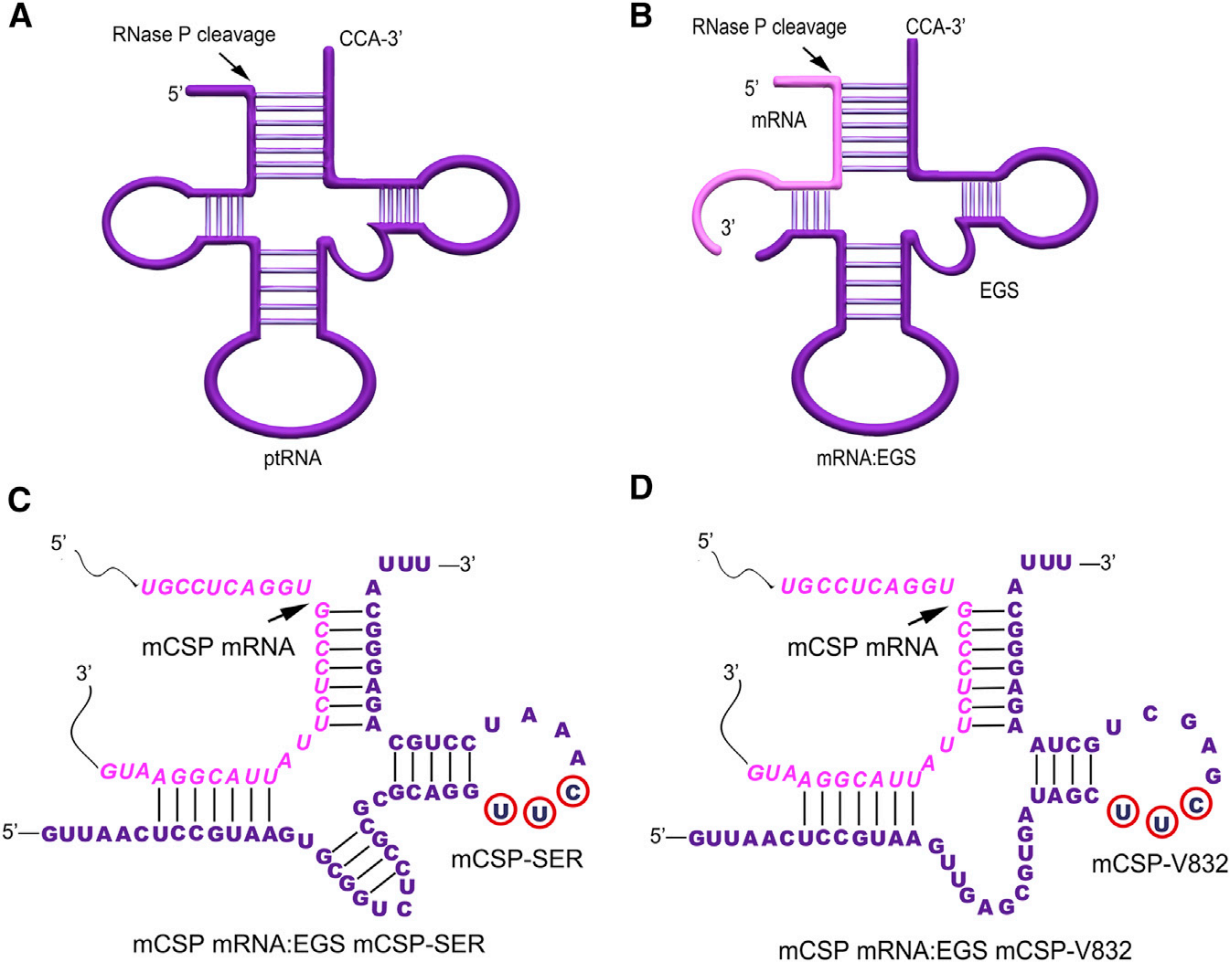
RNase P Substrates (A) A natural precursor to tRNA (ptRNA). (B) A target mRNA (in red) hybridizing to an EGS (in purple). (C and D) An mCSP mRNA sequence (in red) hybridizing to EGS mCSP-SER (C) and mCSP-V832 (D) (in purple). The EGS domain of mCSP-SER and mCSP-V832 originated from tRNA^Ser^ and variant V832, respectively.

Therapeutic RNA- or DNA-based agents, including those used in RNAi and antisense therapy, have given great promise for future treatment of illness.^1^, ^2^ Every method using these agents contains its own strengths and shortcomings regarding potency, possible effects from nonspecific targeting of undesired genes, and challenges in delivering the agents *in vivo*. Ribonuclease P (RNase P) is being developed as a promising gene-targeting agent to regulate expression of mRNAs and proteins.^3^, ^4^ During tRNA maturation, RNase P enzymatically removes the 5′ leader sequence from a precursor to tRNA (pre-tRNA).^3^, ^5^, ^6^ This enzyme catalyzes the hydrolysis of various naturally occurring substrate molecules due to its unique capability to recognize the structural formation of targeted substrates (Figure 1A). In other words, RNase P can recognize and cleave any RNA molecules resembling a tRNA-like complex in which a uniquely engineered external guide sequence (EGS) binds to a target mRNA (Figure 1B)^7^, ^8^. EGSs were demonstrated in various experiments to guide and stimulate RNase P to cleave numerous target mRNAs of different hosts and viruses and suppress the expression of these mRNAs in bacteria and in cultured mammalian cells.^8^, ^9^, ^10^, ^11^, ^12^, ^13^, ^14^

Enhancing RNase-P-mediated cleavage efficiency by developing better EGSs is critical to the usage of EGS-based technology for therapeutic purposes *in vivo*. By applying a selection procedure *in vitro*, novel EGS variants, which were capable of directing RNase-P-mediated cleavage of the thymidine kinase (TK) mRNA of herpes simplex virus 1 (HSV-1) *in vitro* more efficiently than the EGS originating from a naturally occurring tRNA, were identified.^15^ However, whether these EGS variants can be used to suppress expression of viral genes and treat infection in animal models has not been reported.

Human cytomegalovirus (CMV) is a medically important pathogen that causes life-threatening complications in newborns and individuals with a compromised immune system.^16^ In mice, infection and pathogenesis of murine cytomegalovirus (MCMV) share many similar aspects with human CMV in humans and could be used as an animal model to further understand human CMV biology.^17^, ^18^ For instance, CB17 SCID mice, which lack both T and B lymphocytes and are favorably permissive to MCMV infection,^16^, ^19^ can be used to study the progression of CMV infection upon treatment of antivirals in order to develop novel antiviral therapies.

In the study reported here, an EGS was engineered to bind to a shared region of the mRNAs that encode MCMV assemblin and capsid scaffolding protein (mCSP), which are indispensable for capsid formation and MCMV replication.^16^, ^20^ Our experiments revealed that the engineered EGS, mCSP-V832, was better at inhibiting MCMV gene expression and reducing viral replication than mCSP-SER, the EGS originating from a natural tRNA, resulting in a reduction in mCSP expression of more than 92% and 8,000-fold reduced virus production in cultured cells. In MCMV-infected severe combined immunodeficiency (SCID) mice hydrodynamically transfected^21^, ^22^, ^23^ with constructs expressing engineered mCSP-V832, we observed significant decreases in viral gene expression and replication and increases in animal survival. To our knowledge, these experiments show that engineered EGS variants have better efficacy in reducing MCMV gene expression and infection *in vivo* than those derived from a wild-type tRNA sequence. Furthermore, our findings imply the possibility of engineering very potent EGS variants for the treatment of viral infections.

## Results

### RNase-P-Mediated Slicing of MCMV CSP mRNA Sequence Directed by EGSs *In Vitro*

The mRNA coding for MCMV capsid scaffolding protein (mCSP) is completely within and terminates at the identical 3′ poly(A) location with the mRNA coding for viral assemblin.^24^, ^25^ Consequently, EGSs can guide RNase P to cleave at the shared sequences of these two mRNAs. In order to attain the highest efficiency of RNase-P-mediated cutting, we attempted to identify the mCSP mRNA regions that exhibit sequence features important for interactions with RNase P and EGS to achieve efficient cleavage and that are potentially exposed to hybridization of our constructed EGSs. These sequence features include (1) the nucleotides 5′ and 3′ adjacent to the site of cleavage as a pyrimidine and a guanosine, respectively, and (2) an uracil 8 nt downstream of the site of cleavage.^3^, ^26^ A mapping method with dimethyl sulfate (DMS)^27^, ^28^, ^29^ was employed to reveal DMS-modified segments of the mCSP mRNA. In these experiments, we grew MCMV-infected cells in DMS-containing growth media. The mCSP mRNA sequences subjected to DMS modification were determined by primer extension experiments. We designated a site 195 nt downstream of the CSP translational initiation codon^24^, ^25^, ^30^ as the RNase P cutting site. This location happens to be highly exposed to DMS modification and thus probably open to EGS hybridization. This site also has the sequence features important for interactions with RNase P and EGS to achieve efficient cleavage^3^, ^26^ (Figure 1).

In earlier studies, we performed a selection process *in vitro* to identify EGS RNA variants with higher efficiency to induce RNase P to cleave a targeted mRNA than those EGSs originating from a tRNA.^15^ One engineered variant, V832, exhibited one of the best activities in directing RNase P to cut the mCSP and HSV-1 TK mRNAs *in vitro* (see below; Table 1).^15^ In the current study, we investigated the efficacy of V832 in inhibiting MCMV infection in cultured cells and in mice.

**Table 1:** Kinetic Analyses of RNase P Cleavage Reactions for Substrates ptRNA^Ser^ or mCSP mRNA Sequence (ms38) in the Presence of Different EGSs

Substrate	K_m_ (μM)	V_max (apparent)_ (pmol·min^−1^)	V_max(apparent)_/K_m(apparent)_ (pmol·μM^−1^·min^−1^)	K_D_ (μM)
ptRNA^Ser^	0.020 ± 0.005	0.040 ± 0.015	2.0 ± 0.5	
Substrate ms38				
+mCSP-SER	0.60 ± 0.08	0.024 ± 0.010	0.040 ± 0.010	1.9 ± 0.4
+mCSP-SER-C	ND	ND	<0.001	1.9 ± 0.5
+mCSP-V832	0.31 ± 0.08	0.78 ± 0.20	2.5 ± 0.5	0.022 ± 0.004
+mCSP-CV832-C	ND	ND	<0.001	0.023 ± 0.004

Values were derived from experiments that were in triplicate and repeated three times. Experimental details are described in Materials and Methods. ND, not determined.

Construction of functional EGS mCSP-V832, which shares similar structure to a part of a tRNA consisting of a T-stem, a T-loop, and a variable region, was performed by joining the EGS domain of V832 to oligonucleotides that are complementary to the targeted mCSP mRNA region (Figure 1D). We constructed another functional EGS, mCSP-SER, from tRNA^Ser^ similarly (Figure 1C). Control EGSs mCSP-V832-C and mCSP-SER-C were similarly engineered from mCSP-V832 and mCSP-SER, respectively. Compared to mCSP-V832 and mCSP-SER, these two control EGSs had mutations (5′-UUC-3′ → AAG) at the highly conserved region in the T-loop (Figures 1C and 1D). These nucleotides^31^ have been shown to be important for tRNA interaction with RNase P.^3^, ^6^ EGSs with these mutations failed to induce RNase-P-mediated cleavage.^14^, ^32^

Functional EGSs mCSP-SER and mCSP-V832 were found to induce RNase P to cleave substrate ms38 that contained the mCSP mRNA sequence of 38 nt *in vitro* (Table 1). The RNase-P-mediated cleavage efficiency [V_max(apparent)_/K_m(apparent)_] induced by mCSP-V832 was at the minimum 60 times higher than that induced by mCSP-SER, which originated from a natural tRNA (Table 1). The targeting activity of mCSP-V832 increases, probably because of additional EGS-mRNA tertiary interactions that may induce further stabilization of the mCSP-V832-ms38 complex. For this hypothesis to be true, we anticipate that mCSP-V832 may hybridize to ms38 better than mCSP-SER. EGS mCSP-V832 has ∼80 times higher binding affinity (as measured by the dissociation constant [K_D_]) to ms38 than mCSP-SER (Table 1). Since mCSP-V832 and mCSP-SER share identical sequences complementary to ms38 (Figures 1C and 1D), these results raise the possibility that variant mCSP-V832 may have enhanced tertiary interactions with substrate ms38, resulting in better hybridization and stability of the EGS-target complex and leading to more efficient RNase-P-mediated cleavage.

With control EGSs mCSP-SER-C and mCSP-V832-C, RNase-P-associated cleavage of ms38 was rarely observed and was at least 2,500 times slower than that observed for mCSP-V832 (Table 1). CSP-SER-C and mCSP-V832-C shared identical sequences complementary to the mCSP mRNA sequence as mCSP-SER and mCSP-V832 (Figures 1C and 1D) and showed comparable binding affinities (K_D_) *in vitro* to ms38 as mCSP-SER and mCSP-V832, respectively (Table 1). Hence, we used mCSP-V832-C and mCSP-SER-C as controls for the antisense effect of these EGSs.

### Expression of the Engineered EGSs in Tissue Culture Settings

**Figure 2:**
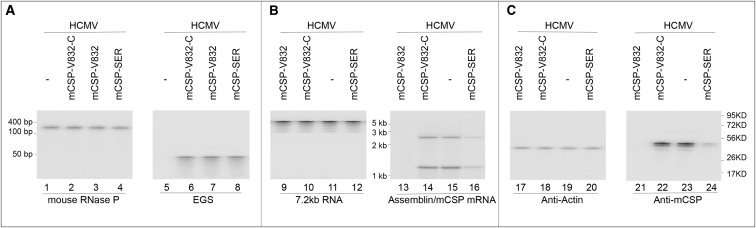
Expression of EGS RNAs, and MCMV mRNAs, and Proteins in Cultured Cells (A) RNA samples (30 μg) from parental NIH 3T3 cells (−) and EGS-expressing cells were separated on 2% agarose gels containing formaldehyde (lanes 1–8). (B) RNA samples (50 μg) were harvested from parental NIH 3T3 cells (−) and EGS-expressing cells infected with MCMV (MOI = 1) at 36 hr postinfection and then separated on 8% denaturing polyacrylamide gels containing urea (lanes 9–16). (C) Protein samples (60 μg) were prepared from parental NIH 3T3 cells (−) and EGS-expressing cells infected with MCMV (MOI = 1) at 48 hr postinfection and separated on 9% SDS polyacrylamide gels. Separated RNA and protein fractions were transferred to nitrocellulose membranes and then reacted with a [^32^P]-radiolabeled probe for the DNA sequences coding for mouse RNase P RNA (lanes 1–4), EGSs (lanes 5–8), MCMV 7.2-kb RNA (lanes 9–12), and mCSP mRNA (13–16) or antibodies against mouse actin (lanes 17–20) and MCMV mCSP (lanes 21–24).

We selected retroviral LXSN vector and U6 RNA promoter to express EGSs.^32^, ^33^ Cell lines expressing mCSP-SER, mCSP-SER-C, mCSP-V832, and mCSP-V832-C were derived from NIH 3T3 cells. We also produced another cell line expressing TK112,^34^ an EGS that targets the HSV-1 TK mRNA. With TK112, we observed no RNase-P-mediated cleavage of ms38 *in vitro* (data not shown). TK112 was selected to examine whether EGS RNA with an improper guide sequence would induce RNase P to cleave the mCSP mRNA sequence. Northern blot analyses were used to quantify EGS RNA expression in each cloned cell line, and expression levels of mouse RNase P RNA served as the loading control (Figure 2, lanes 1–8).^3^, ^35^ Only cell lines expressing the same levels of the EGS RNAs were used for subsequent studies. In our 3-(4,5-dimethylthiazol-2-yl)-2,5-diphenyl tetrazolium bromide (MTT) experiments, the control cells, which only contained the LXSN vector alone, were similar to the EGS-expressing cells with regard to their growth and viability for up to 90 days (data not shown). These studies suggest that EGS expression may not affect cell growth and viability.

### Inhibition of MCMV Gene Expression and Production by EGS in Cultured Cells

To establish how effectively the EGSs can inhibit MCMV gene expression, total RNAs were obtained from cells infected with MCMV (MOI of 1) at 8–72 hr postinfection. Viral 7.2-kb RNA expression^36^, ^37^ was used as the internal control to quantify the levels of mCSP and assemblin mRNAs (Figure 2, lanes 9–12). A decline of 92%–93% and 75%–76% in the expression levels of assemblin and mCSP mRNAs was documented in cells expressing mCSP-V832 and mCSP-SER, respectively (Figure 2, lanes 13–16; Table 2). On the other hand, a decrease in the expression levels of the two mRNAs of less than 10% was documented in cells expressing mCSP-V832-C, mCSP-SER-C, or M1-TK (Figure 2, lanes 13–16; Table 2). Using northern blot analyses and rapid amplification cDNA ends (RACE) PCR assays, we detected no specific products of cleavage of the viral mRNAs by RNase P in these cells, possibly because these cleavage products, which are RNAs lacking either a 5′ cap structure or a 3′ poly(A) sequence, are extremely unstable and quickly hydrolyzed intracellularly.

**Table 2:** Reduction in the Level of Viral Gene Expression in EGS-Expressing Cells and NIH 3T3 Cells

	Viral Gene Class	EGS RNA					
		NIH 3T3	TK112	mCSP-SER-C	mCSP-V832-C	CSP-SER	mCSP-V832
M36 mRNA	α	0%	0%	1%	0%	0%	0%
m44 mRNA	β	0%	0%	1%	1%	1%	1%
mCSP mRNA	γ	0%	0%	5%	6%	76% ± 8%	93% ± 7%
Assemblin mRNA	γ	0%	0%	5%	6%	75% ± 8%	92% ± 8%
mie1 protein	α	0%	0%	1%	1%	0%	0%
M112 protein	β,γ	0%	0%	0%	0%	0%	0%
CSP protein	γ	0%	0%	5%	7%	74% ± 8%	92% ± 8%
Assemblin protein	γ	0%	0%	6%	6%	75% ± 8%	93% ± 9%
M99 protein	γ	0%	0%	0%	1%	1%	0%

The values are derived from experiments that were repeated three times. SDs less than 5% are not shown.

We used western blot experiments to assay MCMV mCSP protein expression (Figure 2, lanes 21–24) with mouse actin as the internal control (Figure 2, lanes 17–20). Viral mCSP protein expression decreased by 92%–93% and 74%–75% in cells expressing mCSP-V832 and mCSP-SER RNA, respectively, and by 10% (or less) in cells expressing mCSP-V832-C or mCSP-SER-C RNA (Table 2). These observations suggest that the substantial decline in the levels of the mCSP mRNA and protein expression in cells expressing mCSP-V832 and mCSP-SER resulted from RNase-P-catalyzed cleavage of the target mRNA directed by the EGSs. The low level of repression in mCSP expression found in cells expressing mCSP-V832-C and mCSP-SER-C likely resulted from an antisense effect. This is because these control EGSs had similar binding affinities to the targeted mRNA as mCSP-V832 and mCSP-SER, respectively, but could not induce RNase-P-mediated cleavage, because there were point mutations in their T-loop segments.

**Figure 3:**
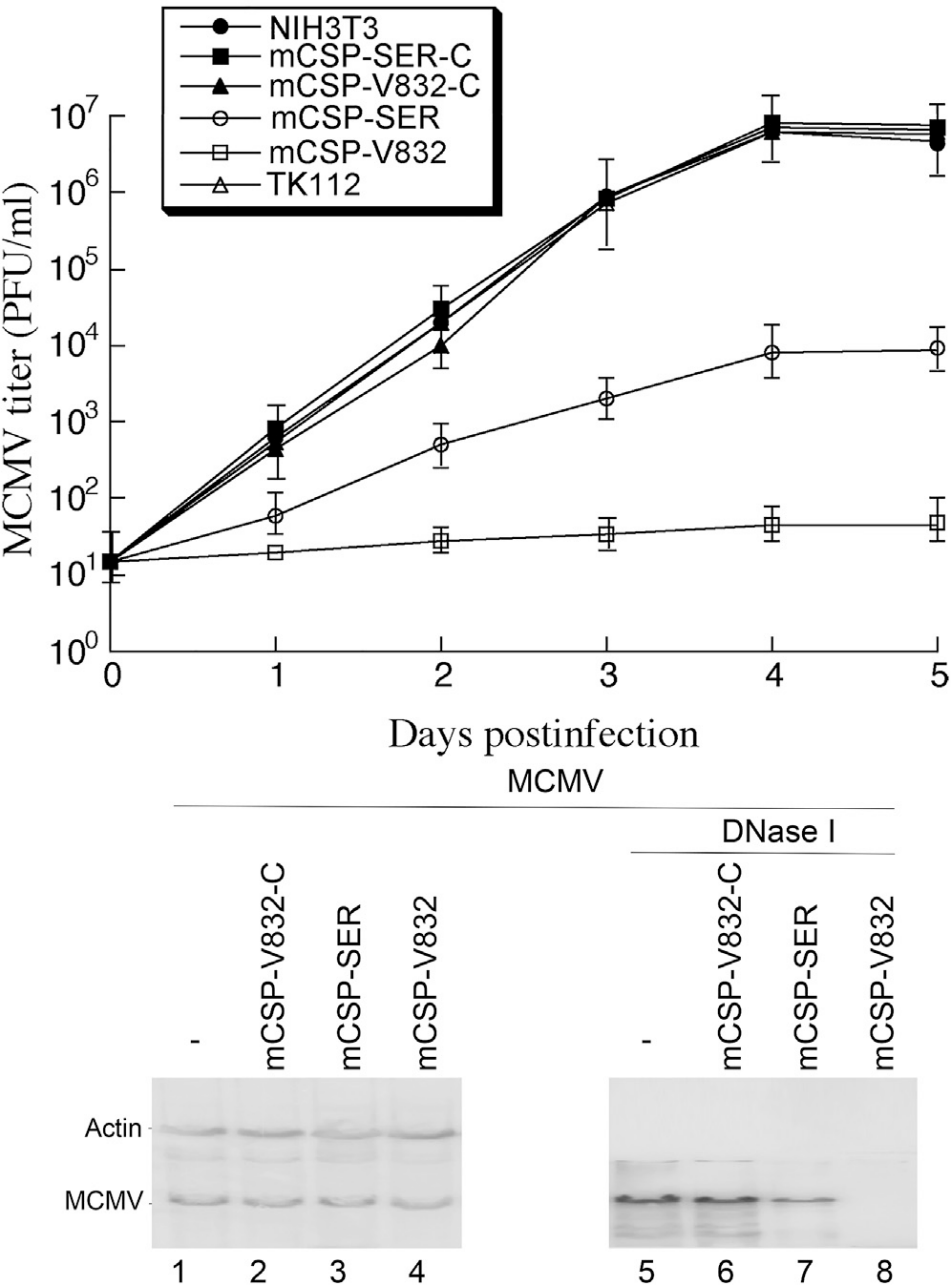
Growth of MCMV and Level of Encapsidated (Lanes 5–8) and Total Intracellular (Lanes 1–4) Viral DNA in Cultured Cells Experiments were performed as described in Materials and Methods. Virus stocks were titered using plaque assays.^19^ SD is indicated by error bars. These values are derived from experiments that were in triplicate and repeated three times. DNA samples (lanes 1–8) were prepared from MCMV-infected cells (MOI of 2) at 48 hr postinfection. Levels of MCMV mie1 sequence were assayed by PCR using mouse actin DNA sequence as internal controls. The [^32^P]-labeled PCR products were separated in nondenaturing gels.

We studied the inhibition of MCMV production in the cells expressing EGSs by infecting cells with MCMV at an MOI of 1. 4 days postinfection, viral production decreased by at least 8,000- and 700-fold in cells that expressed mCSP-V832 and mCSP-SER, respectively (Figure 3). No significant decrease was documented in cells expressing mCSP-V832-C, mCSP-SER-C, or TK112 (Figure 3). Thus, EGSs that inhibit mCSP expression reduce MCMV production and growth.

### Inhibition of MCMV Capsid Formation by EGS RNA-Directed Targeting of mCSP mRNA

Reducing mCSP expression could result in inhibition of MCMV lytic replication, because mCSP is crucial for viral capsid assembly.^24^, ^25^ There is a possibility that the documented decline of viral production in the EGS-expressing cells results not from RNase-P-mediated slicing of the mCSP/assemblin mRNAs directed by the EGSs but from other effects of the EGSs on viral lytic replication, such as inhibition of viral immediate early genes or genomic DNA replication.^16^ To address these issues and study the specificity of EGS-directed RNase P cleavage, two series of experiments were performed to examine the steps of the viral lytic cycle in the EGS-expressing cells and study the antiviral effects of the EGSs.

We assayed MCMV gene expression in EGS-expressing cells in the first series of experiments. Blocking mCSP and assemblin expression is not anticipated to alter the expression of other MCMV genes, including α (immediate-early), β (early), and γ (late) genes, which are not controlled by mCSP or assemblin.^16^ We used northern blot experiments to quantify the levels of viral M36 (an immediate-early transcript) and M44 mRNAs (an early and late transcript). Furthermore, western blot analyses were employed to quantify the protein levels of mie1 (an immediate-early protein), M112 (an early and late protein), and M99 (a late protein). No noteworthy difference in the expression levels of these MCMV genes was observed among EGS-expressing cells and parental NIH 3T3 cells (Table 2). Thus, mCSP-SER and mCSP-V832 expression seemed to hinder the expression of CSP and assemblin explicitly but did not alter the expression of other MCMV genes.

We examined the possibility that MCMV genomic replication and capsid development were altered in the cells expressing mCSP-SER and mCSP-V832 RNAs in the second series of experiments. A semiquantitative PCR assay for the detection of viral mie1 sequence was carried out to determine intracellular level of MCMV DNA in the total DNA samples isolated from infected cells. Levels of mouse actin DNA were used as the internal control (Figure 3, lanes 1–4). The level of intracellular MCMV DNA signifies the production level of the MCMV genome, because the viral DNA genome presents as an episome that is not integrated into mouse chromosomes.^16^ We also examined the level of encapsidated viral DNA in order to study mature MCMV capsid formation within the cells. We used DNase I to treat DNA samples from the lysates of infected cells. The packaged MCMV DNA sequences are not anticipated to be susceptible to DNase I treatment while the DNA sequences that are not “encapsidated” are subjected to DNase I digestion. PCR quantification of the levels of the viral mie1 sequence in samples treated with DNase I was performed to determine the encapsidated MCMV DNA levels (Figure 3, lanes 5–8).

We found no significant difference in the levels of total intracellular (both “packaged” and “un-packaged”) MCMV DNA in the EGS-expressing cells (Figure 3, lanes 1–4). When the samples were first treated with DNase I and then subjected to PCR assays, the levels of “packaged” viral DNA were notably lower in cells expressing mCSP-SER and much lower in cells expressing mCSP-V832 than in cells expressing no EGS or the control EGSs mCSP-SER-C, mCSP-V832-C, or TK112 (Figure 3, lanes 5–8). These findings suggest that inhibiting mCSP and assemblin expression by EGS-directed RNase P cleavage has no effect on the replication of MCMV DNA but results in the blockage of the steps when the capsid is formed. Moreover, the selected EGS variant (i.e., mCSP-V832) had better efficacy in inhibiting HCMV capsid development than the EGS (i.e., mCSP-SER) originating from a natural tRNA. Different from most currently US Food and Drug Administration (FDA)-approved compounds (e.g., ganciclovir), which block HCMV genomic DNA replication,^16^ the EGSs mCSP-V832 and mCSP-SER exhibited a unique mode of antiviral action by blocking HCMV capsid maturation without affecting viral genomic DNA replication. The results presented here further suggest that this unique mode of action can effectively inhibit HCMV infection and progeny production.

### Inhibition of MCMV Infection and Pathogenesis by EGSs in Animals

**Figure 4:**
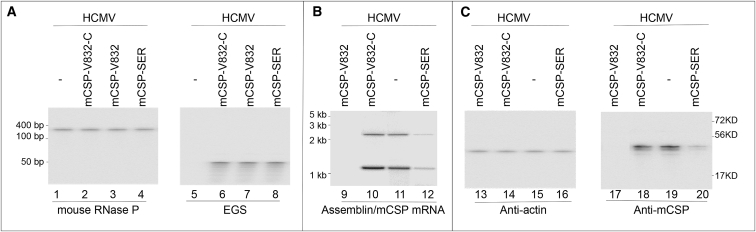
Inhibition of MCMV mRNA and Protein Expression in Mice Transfected with EGS Constructs Expression of EGS (A) and MCMV mRNAs (B) and protein (C) in mice. Experimental details are described in Materials and Methods. RNA (30 μg) and protein (60 μg) samples were isolated from livers of MCMV-infected mice receiving PBS only (−) and PBS containing EGS constructs at 14 days postinfection. RNA samples were separated on 2%-formaldehyde-containing agarose gels (lanes 1–8) or 8%-urea-containing polyacrylamide gels (lanes 9–12). Protein samples were separated on 9% SDS polyacrylamide gels (lanes 13–20). The separated RNA and protein fractions were reacted with a [^32^P]-radiolabeled probe for the DNA sequences coding for mouse RNase P RNA (lanes 1–4), EGSs (lanes 5–8),^3^ and mCSP mRNA (9–12) or antibodies against mouse actin (lanes 13–16) and MCMV mCSP (lanes 17–20).

MCMV infection of immunodeficient SCID mice represents an outstanding model for studying CMV biology and investigating the antiviral activity of various compounds and therapeutic approaches.^19^, ^38^ To study whether EGSs affect MCMV infection *in vivo*, groups of SCID mice (5 animals per group) were infected with MCMV (1 × 10^4^ plaque-forming units [PFU]/animal) and transfected hydrodynamically^21^, ^22^, ^23^ with plasmids carrying LXSN-EGS DNA at 24 hr postinfection. We repeated the transfection every 3 days to provide continuous expression of EGSs. We determined transfection efficiency by assaying the expression of EGS RNAs in the tissues using northern blot analysis with the expression of mouse RNase P RNA as the internal control (Figure 4, lanes 1–8). Furthermore, the transfection efficiency was also assessed by examining the expression of GFP, whose coding sequence is also located in the LXSN vector (data not shown). We found considerable amounts of EGS and a substantial number of cells expressing GFP in the spleens and livers of transfected mice, suggesting that the hydrodynamic transfection procedure was efficient, as previously reported by other investigators^21^, ^22^, ^23^ (Figure 4B; data not shown).

**Figure 5:**
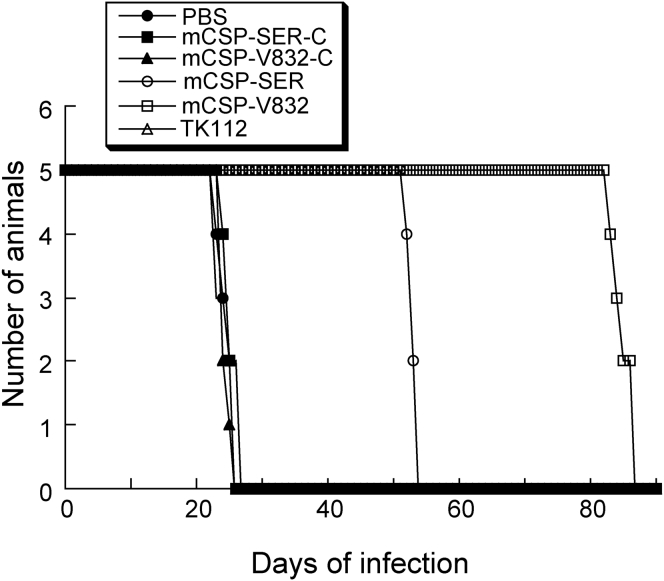
Survival of MCMV-Infected CB17 SCID Mice Hydrodynamically Transfected with PBS Alone or PBS with Different LXSN-EGS DNA Mice (5 animals per group) were infected intraperitoneally by MCMV and transfected hydrodynamically. Experiments were performed as described in Materials and Methods. We assessed animal survival for at least 90 days postinfection.

We performed three series of experiments to understand the effects of EGSs on MCMV virulence and infection *in vivo*. In the first series of experiments, we measured the survival rates of the mice transfected with the mCSP-V832 and mCSP-SER plasmids in comparison with those injected with only PBS or PBS with plasmid constructs containing the sequences of the control EGSs mCSP-V832-C, mCSP-SER-C, or M1-TK. All uninfected mice hydrodynamically transfected with EGS-LXSN survived and showed no adverse symptoms for at least 3 months. Similar results were also found in untreated and uninfected mice (data not shown). The survivability of MCMV-infected animals transfected with the mCSP-V832-C, mCSP-SER-C, or M1-TK plasmids was similar to that of animals receiving only PBS, as all of these infected animals died within 26–27 days postinfection (Figure 5). On the other hand, the survivability of infected animals that expressed mCSP-V832 and mCSP-SER enhanced drastically, as no mice died until 80 and 50 days postinfection, respectively (Figure 5).

**Figure 6:**
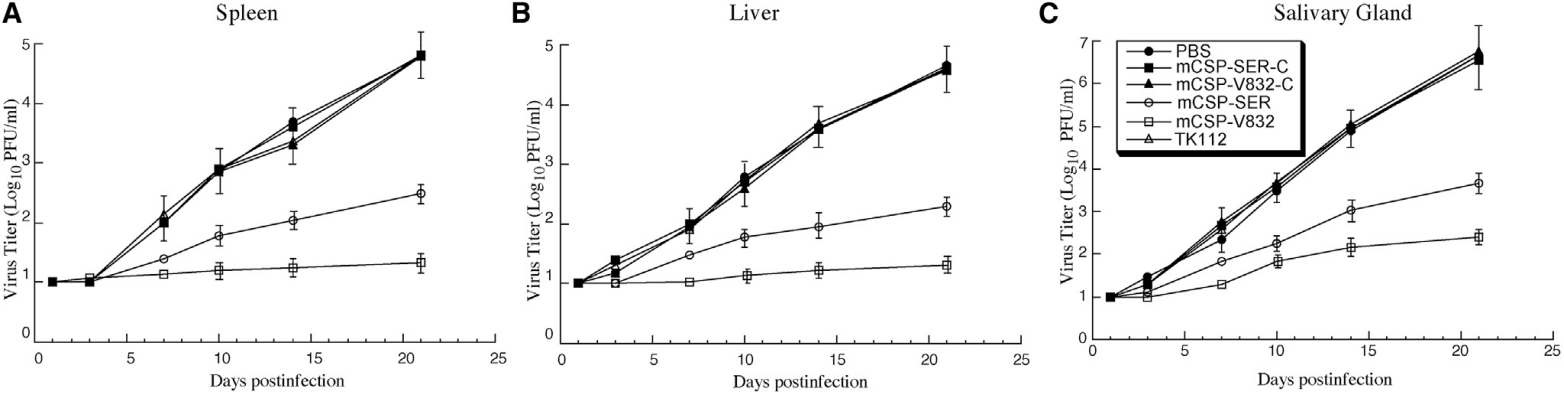
Inhibition of MCMV Growth in Mice Transfected with EGS Constructs Titers of MCMV in spleen (A), liver (B), and salivary glands (C) of MCMV-infected CB17 SCID mice hydrodynamically transfected with PBS alone or PBS with different LXSN-EGS DNA. Experimental details regarding the infection and hydrodynamic transfection of animals are described in Materials and Methods. Different tissues were harvested from animals at 1, 3, 7, 10, 14, and 21 days postinfection. Viral titers in the tissues were determined by plaque assays. The limit of detection was 10 PFU/mL of the tissue homogenate. Values were derived from experiments that were in triplicate andrepeated three times.

In the second series of experiments, we examined MCMV growth in various organs of mice during a 3-week infection phase prior to the onset of death of the infected mice in order to understand the benefits or consequences of EGS expression in SCID mice. At 1, 3, 7, 10, 14, and 21 days postinfection, we collected the spleens, livers, and salivary glands from euthanized animals and assessed viral infection and production by determining virus titers in these tissues.^19^, ^38^ The viral titers in each of the organs from mice injected with control EGS (i.e., mCSP-V832-C, mCSP-SER-C, or M1-TK) constructs were identical to those in the same organs from mice injected with only PBS, suggesting that these control EGSs do not affect MCMV infection and production *in vivo* (Figures 6A–6C). On the other hand, the titers of MCMV in the organs from mice injected with the functional EGS (i.e., mCSP-V832 and mCSP-SER) constructs were steadily lower than those from mice injected with only PBS during the entire 21-day infection phase. At 3 weeks postinfection, the MCMV titers in the spleens, livers, and salivary glands of the mCSP-V832-injected mice were lower than those from mice injected with PBS only by 2,500-, 2,000-, and 20,000-fold, respectively (Figure 6). The MCMV titers in the spleens, livers, and salivary glands of the mCSP-SER-injected mice were lower than those of mice injected with PBS only by 200-, 250-, and 1,500-fold, respectively (Figure 6). These observations imply that mCSP-V832 and mCSP-SER expression reduces MCMV infection and production *in vivo*.

In the third series of experiments, we studied MCMV gene expression in the organs. At 2 weeks postinfection, we detected substantial levels of MCMV assemblin/mCSP mRNAs and mCSP protein in the livers of the animals expressing control EGSs mCSP-V832-C, mCSP-SER-C, and TK112 (Figure 4, lanes 9–20). However, we detected no noticeable expression of assemblin/mCSP mRNAs and mCSP protein in mCSP-V832- and mCSP-SER-treated animals at the same time points (Figure 4, lanes 9–20; data not shown). Notably, we observed no difference in the expression of mCSP mRNA and protein between mice injected with PBS only and those transfected with plasmids containing control EGSs mCSP-V832-C, mCSP-SER-C, or TK112 sequences (Figure 4, lanes 9–20). These findings indicate that viral infection is repressed in mice treated with mCSP-V832 and mCSP-SER constructs.

## Discussion

The technology based on RNase-P-associated EGS signifies an appealing method for therapeutic application.^3^, ^4^ Nevertheless, the knowledge regarding the rate-limiting step of EGS-based targeting in tissue culture and in animals and how to improve the efficiency of EGS-based technology is currently limited. In this report, an EGS was engineered to target an accessible region of the MCMV mCSP mRNA that contained sequence features important for interaction with EGS and RNase P to achieve efficient cleavage. We hypothesize that the efficacy of the EGS-based gene targeting approach in tissue culture and in animals is regulated by the overall catalytic efficiency (i.e., V_max_/K_m_) of the RNase P in hydrolyzing the target mRNA induced by the EGS. For this hypothesis to be true, an increase in the efficiency of RNase-P-associated cleavage mediated by EGS should result in better inhibitory effects on targeted mRNA expression in cultured cells and living organisms such as mice.

Our study provides direct evidence that an engineered EGS selected *in vitro* (i.e., mCSP-V832) is ∼60-fold more active [V_max(apparent)_/K_m(apparent)_] in guiding RNase P to produce a cleavage in the mCSP mRNA sequence *in vitro* than the EGS (i.e., mCSP-SER) originating from the naturally occurring tRNA^Ser^ sequence. The variant mCSP-V832 seemed to be better in reducing mCSP/assemblin expression and viral replication in tissue culture and mice than mCSP-SER. On the other hand, we documented a decrease of less than 10% in mCSP/assemblin expression and MCMV replication in cells and mice expressing control EGSs mCSP-SER-C, mCSP-V832-C, or TK112. EGSs mCSP-SER-C and CSP-V832-C had binding affinities to ms38 comparable to mCSP-SER and mCSP-V832, respectively (Table 1). However, they could not direct RNase P to cleave the substrate, because the nucleotide changes at their T-loop regions prevented RNase P interaction (Figure 1; Table 1). These observations indicate that the detected suppression of viral gene expression and replication in cells and mice expressing mCSP-V832 and mCSP-SER can be credited to RNase-P-mediated cleavage of the target mCSP mRNA stimulated by the two EGSs as opposed to the antisense effect or additional nonspecific effects from the EGSs. Likewise, the experiments indicate that mCSP-V832, which has better activity [i.e., V_max(apparent)_/K_m(apparent)_] to stimulate RNase P-associated cleavage of mCSP mRNA sequence *in vitro*, is also more effective in blocking MCMV gene expression and replication in tissue culture and mice than mCSP-SER. Our findings support the notion that increasing the efficiency of EGS to induce RNase P to cleave a target mRNA may help enhance the effects on inhibition of gene expression in tissue culture and in animals *in vivo*.

The results presented in this study imply that the EGS variants are stably expressed and active in blocking gene expression *in vivo* and that blockage of MCMV mCSP expression and infection in mice is the direct consequence of the activity of the EGSs to direct RNase P to hydrolyze the mCSP mRNA. First, we could detect noticeable levels of EGS RNAs in cultured cells and different organs of mice such as livers and spleens. Second, in our MTT experiments, we found no differences in growth and viability between cell lines expressing engineered EGSs and parental cells for up to 90 days. After hydrodynamic transfection of the LXSN-EGS plasmids, uninfected mice showed no adverse symptoms for 3 months compared to the mice that received PBS only (data not shown). These observations suggest that expressing these EGSs may be non-toxic to cells and animals. Third, EGS RNAs specifically repressed mCSP/assemblin expression. Expression levels of other MCMV genes such as M36, mie1, M44, M112, and M99 were not altered in cells that expressed the EGSs (Table 2). Furthermore, EGSs seemed to be functional in guiding RNase P to cleave target mCSP mRNA in mice. Decreased mCSP expression, reduced viral titers, and enhanced survivability were documented in mice receiving mCSP-V832 and mCSP-SER plasmids, but not in mice receiving PBS only or PBS with control EGS mCSP-SER-C, mCSP-V832-C, or M1-TK constructs. These findings suggest that EGSs effectively direct RNase P to cleave the target mCSP mRNA *in vivo*, leading to decreased mCSP expression, reduced MCMV production, and improved survivability in the infected animals.

HCMV infection can be devastating and fatal to individuals with immune system deficiencies, such as recipients of organ transplants and AIDS patients. MCMV infection of SCID mice represents a desirable animal model in which to assess CMV pathogenesis in hosts^16^, ^39^, ^40^, ^41^ and assay the efficacy of new antiviral therapeutics.^17^, ^18^ Hydrodynamic transfection via tail vein injection has been demonstrated to transfer plasmid DNA to the livers and spleens with high speed and efficiency.^21^, ^22^, ^23^ Although it is not applicable clinically, the hydrodynamic transfection technique is valuable to validate feasibility of delivering innovative therapeutics into animals and assess their functionality and potency *in vivo*.^21^, ^22^, ^23^ As a herpesvirus,^42^ CMV is known to establish latent infection in bone marrow progenitor cells and create devastating consequences by spreading to other tissues from the bone marrow.^17^, ^18^ One potential anti-CMV approach is to transfer EGS expression cassettes to the progenitor cells associated with the bone marrow, such as CD34^+^ cells, and express EGSs to control viral infection in these cells and *in vivo*.

Compared to other nucleic-acid-based gene-interfering approaches such as antisense oligonucleotides and RNAi, the EGS-based technology is unique, as this method induces RNase P to cleave the target mRNAs. In an elegant study by Stein and colleagues, RNase-P-mediated cleavage is specific and does not generate non-specific “irrelevant cleavage” that is associated with RNase-H-mediated cleavage induced by antisense phosphorothioate oligonucleotides.^10^ Furthermore, recent studies, as well as this study, show that EGSs effectively block gene expression in both cultured cells and animals and that they may be as effective as small interfering RNAs (siRNAs) and ribozymes in knocking down gene expression.^8^, ^9^, ^10^, ^11^, ^12^, ^13^, ^14^ Thus, EGSs represent a new and promising class of nucleic-acid-based gene-interfering reagents for therapeutic application. Further studies on the effectiveness of the EGS-based approach and other nucleic-acid-based gene-interfering approaches should reveal the advantages and shortcomings of the EGSs compared to other DNA/RNA-based gene targeting molecules. Because CMV is a DNA virus, genome editing approaches such as the transcription activator-like effector nuclease (TALEN) and CRISPR/CAS9 systems may be advantageous by targeting the viral genomic DNA.^43^, ^44^ However, the efficacy of these genome editing approaches in reducing viral genomic DNA levels has not yet been determined,^45^ while knocking down viral essential mRNA expression by numerous methods such as RNAi and RNase P has been shown to yield impressive inhibition of viral infection and replication.^1^, ^2^, ^3^, ^4^ Further studies to evaluate the effectiveness of both genome editing approaches and mRNA-targeting strategies should facilitate the development of these methods for gene-targeting applications.

Little is known about the toxicity of the EGS molecules. Our results presented in this study suggest that expression of EGSs in cultured cells and mice does not significantly affect the viability of the cells and animals. Further studies on the toxicity of the EGSs are needed in order to determine whether EGS molecules exhibit significant cytotoxicity. Equally elusive is the sequence specificity and off-target effects of the EGS-based technology. The EGS targeting specificity is determined by two different interactions between the EGS and target mRNA.^3^, ^4^ The first interaction is the base-pairing between the target mRNA and the antisense domain of the EGS. The second interaction is between the mRNA and RNase P recognition domain of the EGS (e.g., T-loop). This second interaction stabilizes the mRNA-EGS complex to fold into a tRNA-like molecule. It is conceivable that modulating the second interaction would potentially enhance the sequence specificity of the EGS-based technology and reduce potential off-target effects. Using northern blot analyses and RACE PCR assays, we detected no specific products of RNase P cleavage of viral mRNAs in these cells, possibly because these cleavage products, which are RNAs lacking either a 5′ cap structure or a 3′ poly(A) sequence, are extremely unstable and quickly hydrolyzed intracellularly. Additional studies on these issues will reveal the action and sequence specificity of the EGS technology.

*In vitro* selection allows us to engineer functional RNA molecules that have enhanced activity.^46^, ^47^, ^48^ Additionally, this technique has been applied to give rise to EGSs capable of inducing RNase-P-mediated cleavage of various mRNAs.^15^, ^32^ However, there was no report using the selected EGS variants to modulate gene expression in animals. In this study, EGSs selected *in vitro* with enhanced targeting activity also displayed superior efficacy in suppressing MCMV mCSP and assemblin expression and viral production in tissue culture and in mice. Hence, our study offers a way for the manufacturing of potent EGSs by taking advantage of a selection procedure and optimizing EGS to hybridize with designated mRNAs. Additional research involving the use of EGS in animals will expedite the development of better EGS-based therapeutics.

## Materials and Methods

### Ethics Statement

We performed the study strictly according to the recommendations in the *Guide for the Care and Use of Laboratory Animals* from the NIH. The experimental procedures for all animal studies were approved either by the Animal Care and Use Committee of the University of California, Berkeley (protocol R240) or by the Animal Care and Use Committee of the College of Life Sciences and Technology, Jinan University (Guangzhou, China). All efforts were made to mitigate any suffering experienced by the animals.

### EGS Studies *In Vitro*

We generated the EGS coding sequences by PCR using pV832 and pTK112^34^ as the templates according to previously published methods.^15^, ^34^ The 5′ PCR primer for mCSP-SER and mCSP-SER-C was SER-5 (5′-GGAATTCTAATACGACTCACTATAGGTTAACTCCGTAAGTGCGGTCTCCGCGC-3′). The 3′ primers for mCSP-SER and mCSP-SER-C were SER-3 (5′-AAGCTTTAAATGCCCTCTGCAGGATTTGAACCTGCGCGCGGAGACCGCAC-3′) and SER-C-3 (5′-AAGCTTTAAATGCCCTCTGCAGGATTTCTTCCTGCGCGCGGAGACCGCAC-3′), respectively. The 5′ primer for CSP-V832 and CSP-V832-C was V832-5 (5′-GGAATTCTAATACGACTCACTATAGGTTAAC TCCGTAA GTTGAGCGTGA-3′). The 3′ primers for mCSP-V832 and mCSP-V832-C were V832-3 (5′-AAGCTTTAAATGCCCTCTTAGCAGCTCGAA GCTATCACGCTCAA-3′) and V832-C-3 (5′-AAGCTTTAAATGCCCTCTTAGCAGCTCCTT GCTATCACGCTCAA-3′), respectively. We generated the DNA sequence coding substrate ms38 by PCR using pGEM3zf(+) as a template with the 5′ primer ms38-5-AF25 (5′-GGAATTCTAATACGACTCACTATAG-3′) and the 3′ primer ms38-3 (5′-CGGGATCCGCATTCCGTAATAAGAGGGCACCTGAGGCACCTATAGTGAGTCGTATTA-3′).

Mouse RNase P was obtained from lysates of NIH 3T3 cells as previous noted.^14^, ^32^ The EGS and [^32^P]-labeled MCMV mCSP mRNA substrate was mixed with mouse RNase P at 37°C in buffer A, which comprised 50 mM Tris (pH 7.4), 100 mM NH_4_Cl, and 10 mM MgCl_2_. We then employed electrophoresis to separate the reaction mixtures with denaturing gels and used a STORM840 phosphorimager to analyze the results. Experiments to obtain the values of V_max_ and K_m_ were assayed under multiple turnover settings as noted earlier.^14^, ^15^, ^32^

The equilibrium dissociation constants (K_D_) of EGSs and substrate complexes were determined using an electrophoretic mobility shift assay as described previously.^49^ Binding assays were performed in buffer B (50 mM Tris [pH 7.5], 100 mM NH_4_Cl, 10 mM MgCl_2_, 3% glycerol, 0.1% xylene cyanol, and 0.1% bromophenol blue). We determined the value of K_D_ from a plot of the percentage of product bound versus EGS concentration.^14^, ^15^, ^32^ The binding and cleavage reactions were assayed in triplicate and repeated three times.

### Cells Expressing EGSs

NIH 3T3 cells were transfected with retroviral vector LXSN-EGS DNAs and then incubated with neomycin (500 μg/mL) to produce cloned EGS-expressing cell lines, and the expression of EGS and mouse RNase P RNAs was assayed using northern blot experiments following previously described protocols.^14^, ^15^, ^32^

We performed MTT assays (Sigma) to study cytotoxicity of the EGS expression. At different periods of time, we added MTT (Sigma) (5 mg/mL in PBS) to cells grown in 96-well plates and examined cell viability. We assayed the absorbance at 570 nm on a microplate reader. We carried out experiments in four wells and repeated them three times. With a Nikon TE300 microscope, we also studied the morphology of the cells during the experiments.

### Levels of Viral mRNA and Protein

In northern blot experiments, total RNA fractions were collected from cells and tissues, separated in 1% agarose gels containing formaldehyde, moved to a nitrocellulose film, and hybridized with [^32^P]-labeled DNA probes containing the MCMV DNA sequence or the DNA sequence coding for mouse RNase P RNA.^14^, ^15^, ^32^ The images were captured for analysis using a STORM840 Phosphorimager.

In western blot experiments, the polypeptide samples were collected from cells and tissues, separated on SDS/9% polyacrylamide gels cross-linked with N,N′′methylenebisacylamide, moved to a nitrocellulose film, and detected with the antibodies against MCMV proteins and mouse actin in the presence of a chemiluminescent agent using a STORM840 phosphorimager.^14^, ^15^, ^32^ We performed assays in triplicate, and each assay was repeated three times.

### Levels of Viral DNA

Cells (5 × 10^5^) cultured in 6-well plates were mock-infected or infected with MCMV following previously described procedures.^19^ At 48–96 hr postinfection, total and encapsidated (DNase-I-treated) DNA was prepared as described previously and used as templates for PCR.^50^ The levels of MCMV DNA were measured using a semiquantitative PCR assay with the amplification of the viral mie1 sequence and using the mouse β-actin sequence as the internal control.^50^, ^51^, ^52^ The primers and reaction mixture protocols have been described previously.^50^, ^51^, ^52^ The PCR reaction had 20 cycles with a denaturing step at 94°C for 1 min, followed by an annealing step at 47°C for 1 min and an amplification/extension step at 72°C for 10 min. The PCR experimental conditions were adjusted to guarantee that the amplification was within the linear range. PCR products were amplified with α-[^32^P]-dCTP, subjected to separation on polyacrylamide gels, and detected using a STORM840 phosphorimager.^50^ A standard curve was generated by assaying different concentrations of a template DNA. The plot for MCMV and actin DNA versus dilutions of DNA did not reach a plateau, and the ratios of MCMV DNA to actin DNA were similar among the DNA dilutions, suggesting that the semiquantitative PCR assay is reproducible and accurate.^50^, ^51^, ^52^ PCR results were from experiments that were performed in triplicate and repeated three times.

### Studies in Animals

MCMV (Smith strain) (1 × 10^4^ PFU/animal) was used to infect sets of 4- to 6-week-old CB17 SCID mice (at least five animals per set) (Jackson Laboratory, Bay Harbor, ME) intraperitoneally. At 2 days postinfection, we hydrodynamically transfected different constructs to these mice using previously described protocols.^21^, ^22^, ^23^ We used 24G–27G needles to inject 1–2 mL PBS (based on the ratio of injection volume to body weight) in the absence or presence of 20 μg LXSN-EGS DNA within 5–10 s.^21^, ^22^, ^23^ We performed hydrodynamic transfection every 3 days after the first injection. Transfection efficiency was determined by assessing the expression of EGS RNAs within the organs (e.g., livers) with northern blot experiments and using fluorescent microscopy to survey GFP expression in cells transfected with EGSs.

For studies of viral virulence, we observed mice 2 times a day and documented mortality of infected mice for more than 3 months to determine survival rates.^19^ To analyze MCMV growth in the animals, sets of mice (at least five mice per set) were euthanized at 1, 3, 7, 10, 14, and 21 days postinfection. We collected the organs (i.e., spleens, livers, and salivary glands) and subjected the samples to sonication to create suspension samples that contained DMEM and 10% non-fat milk. We determined the titers of viral samples using plaque assays following procedures published elsewhere.^19^ To assay viral gene expression, total RNA and protein samples were prepared from homogenized tissue samples and analyzed by northern and western blot experiments.^14^, ^19^, ^32^

### Analysis of MCMV Growth with Plaque Assays

We collected tissues and cells, transferred them to 10% skim milk, and subsequently used sonication to generate virus stocks. Virus stocks were assayed with plaque assays in NIH 3T3 as explained earlier.^19^ Titers were derived from experiments that were in triplicate and repeated three times. We documented MCMV titers as PFU/mL homogenized tissues. The detection limit of homogenized tissue was 10 PFU/mL. We assigned a titer of 10 (10^1^) PFU/mL for the samples that were negative at a 10^−1^ dilution.^19^

## Author Contributions

W.L, J.S., M.X., G.-P.V., Z.Y., Y.L., X.S., P.T., S.L., and F.L. conceived and designed the experiments. W.L., J.S., M.X., G.-P.V., Z.Y., Y.L., X.S., and P.T. performed the experiments. W.L., J.S., M.X., G.-P.V., Z.Y., X.S., P.T., S.L., and F.L. analyzed the data: W.L., J.S., M.X., G.V., Z.Y., Y.L., and X.S. contributed reagents, materials, and analysis tools. W.L., J.S., G.-P.V., Z.Y., P.T., S.L., and F.L. wrote the paper. All authors reviewed the manuscript.

## Conflicts of Interest

Z.Y. and X.S. are employees of Jiangsu Affynigen Biotechnologies, Inc. and Guangzhou Qinheli Biotechnologies, Inc.
